# Associations of physical activity intensity with incident cardiovascular diseases and mortality among 366,566 UK adults

**DOI:** 10.1186/s12966-022-01393-y

**Published:** 2022-12-13

**Authors:** Xuanwen Mu, Shuangyan Liu, Mingjian Fu, Mengyun Luo, Ding Ding, Liangkai Chen, Kuai Yu

**Affiliations:** 1 Department of Occupational and Environmental Health, Key Laboratory of Environment and Health, Ministry of Education and State Key Laboratory of Environmental Health (Incubating), School of Public Health, Tongji Medical College, Huazhong University of Science and Technology, Wuhan, 430030 China; 2 Sydney School of Public Health, Faculty of Medicine and Health, the University of Sydney, Camperdown, NSW 2006 Australia; 3 Charles Perkins Center, the University of Sydney, Camperdown, NSW 2006 Australia; 4 School of Public Health, School of Medicine, Shanghai Jiao Tong University, 200240 Shanghai, People’s Republic of China; 5 Department of Nutrition and Food Hygiene, School of Public Health, Tongji Medical College, Huazhong University of Science and Technology, Wuhan, 430030 China

**Keywords:** Physical activity intensity, Incident CVD, All-cause mortality

## Abstract

**Background:**

The associations of the proportion of vigorous physical activity (VPA) to moderate to vigorous physical activity (MVPA) with incident cardiovascular disease (CVD) and all-cause mortality are unclear.

**Methods:**

The present study included 366,566 participants (aged 40–69 years) without baseline CVD from the UK biobank during 2006 to 2010. Cox regression was used to calculate hazard ratios (HRs) and 95% confidence intervals (CIs) for risks of outcomes.

**Results:**

During a median 11.8 years of follow-up, among 366,566 participants (mean age [SD]: 56.0 [8.1]), 31,894 incident CVD and 19,823 total deaths were documented. Compared with no VPA, 0%-30% of VPA to MVPA was associated with 12% and 19% lower risks of incident CVD (HR, 0.88 [95% CI, 0.86–0.91]) and all-cause mortality (HR, 0.81 [95% CI, 0.78–0.84]), respectively. Furthermore, we found that the maximum reduction of risks of incident CVD and all-cause mortality occurred at performing approximately 30% of VPA to MVPA (*P* < 0.001). Compared with participants reporting the lowest levels of MVPA (moderate physical activity [MPA], 0–150 min/week; VPA, 0–75 min/week), those performing 150–300 min/week of MPA and ≥ 150 min/week of VPA experienced the lowest risk of incident CVD (HR, 0.87 [95% CI, 0.79–0.95]) and all-cause mortality (HR, 0.71 [95% CI, 0.63–0.80]). Interestingly, we found that smokers yielded more cardiovascular benefits than non-smokers by performing a higher volume of VPA.

**Conclusions:**

Comparing with UK adults reporting no VPA, engaging in 30% of VPA was associated with the lowest risk of incident CVD and all-cause mortality.

**Supplementary Information:**

The online version contains supplementary material available at 10.1186/s12966-022-01393-y.

## Background

Physical inactivity is a leading risk factor of cardiovascular disease (CVD) and premature death worldwide [1–3]. Current guidelines on physical activity are developed based on the assumption that the same duration of vigorous physical activity (VPA, ≥ 6 metabolic equivalent tasks [METs], such as running) could convey twice the health benefits of moderate physical activity (MPA, 3–5.9 METs, such as brisk walking); the World Health Organization recommended 150–300 min/week of MPA or 75–150 min/week of VPA, or equal combinations [4]. Recent studies have reported that VPA might confer greater benefits in lowering mortality risks than MPA when were conducted at an equivalent amount [5, 6]. A prospective cohort study of 403,681 US adults suggested that VPA was associated with 15% lower all-cause mortality compared with MPA [5]. Another cohort study of 64,913 UK adults also found VPA was associated with 16% lower risk of all-cause mortality versus MPA. However, there were two other studies reported no significant difference in mortality risk were observed among participants performed MPA and VPA [7, 8]. Therefore, more studies are still needed to examine the independent association of VPA in comparison with MPA with mortality risks.

Furthermore, current studies [5–7, 9] drew inconsistent conclusions on the association of VPA versus MPA with CVD, the leading cause of mortality worldwide which accounted for 32% of global deaths in 2019 [10]. The US study found that performing more than 50% to 100% of VPA to total MVPA was associated with a 17% lower risk of CVD mortality [5]. However, the UK study did not observe a significant association of higher VPA with CVD mortality [6]. Despite the mixed findings, these studies only focused on CVD mortality, and to date, no study has examined the association of the proportion of VPA to MVPA with incident CVD, as well as its major subtypes (coronary heart disease [CHD], heart failure [HF] and stroke). In addition, previous studies concerning the association between physical activity and CVD usually focused on total amount of MVPA [11, 12], the independent and joint association of MPA and VPA with health outcomes remained largely unclear. Finding the optimal combination of MPA and VPA would benefit in refining the future guideline on physical activity.

Using data from a prospective cohort of 366,566 UK adults, the current investigation aimed to examine the association of the proportion of VPA to MVPA with risks of CVD (overall and major subtypes), and all-cause mortality, to explore the joint effect of MPA and VPA on these risks, with the hypothesis that higher proportion of VPA to MVPA were associated lower risks of incident CVD and all-cause mortality.

## Methods

### Study population

Our analysis was based on data from the UK Biobank study [13]. Briefly, UK Biobank is a large population-based, prospective cohort study that recruited 502,505 participants aged 40–69 years in the UK from 2006 to 2010 (protocol available at https://www.UKbiobank.ac.UK/key-documents/). All participants provided questionnaire information, physical measurements, and biological samples at the baseline. UK Biobank was approved by the North West Multi-Center Research Ethical Committee (REF: 11/NW/03820). All participants gave written informed consent before enrolment in the study, which was conducted in accordance with the principles of the Declaration of Helsinki.

Among the 502,505 participants recruited at baseline, the present study excluded participants who have withdrawn from the UK Biobank (*n* = 46), pregnant women (*n* = 371), those with baseline CVD (*n* = 36,449), and those with missing information on physical activity (*n* = 91,624) or reported no physical activity (*n* = 7,449), leaving 366,566 participants for the final analytic sample (see Additional file 1). Baseline CVD was ascertained by self-reported information and hospital records [14]. The distribution of baseline characteristics was comparable among participants before and after exclusion (see Additional file 2).

### Physical activity assessment

Data on physical activity was obtained through questionnaires adapted from the IPAQ short version (https://biobank.ndph.ox.ac.uk/showcase/browse.cgi?id=1008&cd=browse), which was reported to have acceptable test–retest reliability and criterion validity in the UK population [15]. Accordingly, we multiplied the energy expended for a specific category of activity (MET: 3.3 for walking, 4.0 for moderate activity and 8.0 for vigorous activity) [15, 16] by the corresponding frequency (times/week) and duration (minutes/time) and summed the corresponding amount to estimate MET-minutes/week of total MVPA. In the present study, we further defined MPA as physical activity of 3.0–5.9 METs therefore MET-minutes/week of walking and moderate activity were summed as the total amount of MPA, and VPA as physical activity of 6.0 METs or more such as running [15, 16]. Among participants with any MVPA, the proportion of VPA to MVPA was calculated by dividing the amount of VPA by the amount of MVPA. The proportion of VPA to MVPA was categorized into one of the three categories: 0% (no VPA), > 0% to ≤ 30%, and > 30%. The 30% cut-off was chosen according to a previous study, which showed a maximum reduction in risk of all-cause mortality appeared at 30% of VPA to MVPA [17].

### Covariates

The present study included demographic characteristics (age, sex [male or female]), sociodemographic characteristics (education [college/university or below college], income [< 18,000 or 18,000–52,000 or ≥ 52,000 ￡/year] [18], race [white or others], Townsend index), lifestyle factors (smoking status [never or current or former], alcohol consumption [0 or 0.1–30 or ≥ 30 g/day] [19, 20], diet quality score [see Additional file 3], sedentary behavior [hours/day] and MVPA [MET-minutes/week]), body mass index (BMI, kg/m^2^) and family history of CVD (yes or no) as covariates according to previous studies on similar topic [5, 7, 17]. Missing indicator approach was used in the regression model by coding missingness as a dummy variable.

### Ascertainment of outcome

UK Biobank data are linked to Hospital Episode Statistics (HES; hospital diagnoses from the National Health Service) and the UK Biobank Cause of Death Registry [21]. Incident CVD and mortality were determined based on the International Classification of Diseases edition 10 (International Classification of Diseases, ICD-10) [22]. HES and mortality data were updated up until December 31, 2020. The primary outcome was incident CVD (ICD-10: I00-I99), its main subtypes including CHD (I20-25), heart failure (HF; I50, I500, I501, I509), and stroke (I60, I61, I63, I64). The secondary outcome was all-cause mortality, its main components including CVD mortality.

### Statistical analysis

Standardized difference was calculated to compare the baseline characteristics across three groups of VPA to MVPA (0%, > 0% to ≤ 30% and > 30%) [23]. A standardized difference less than 0.20 indicates a small difference of characteristic across different groups of VPA to MVPA (0%, > 0% to ≤ 30% and > 30%) [24]. Cox proportional hazard models were used to calculate the hazard ratios (HRs) and 95% confidence intervals (CIs) for incident CVD and all-cause mortality according to three groups of VPA to MVPA. Models were adjusted for age, sex, education, income, race, Townsend index, smoking status, alcohol consumption, sedentary behavior (hours/day), MVPA (MET-minutes/week), BMI (kg/m^2^), diet quality score, and family history of CVD. To test for *P* trend, participants were assigned to the median value of each group of VPA to MVPA (0%, > 0% to ≤ 30% and > 30%), and then this continuous variable was entered into the Cox model [25]. Comparison between adjusted HRs for the first half of follow-up years and for the subsequent years revealed no evidence of departure from the proportional hazards assumption for main analyses.

In order to explore the potential interaction, stratified analyses were performed by socio-demographic characteristics (age, sex, education, Townsend index), lifestyle risk factors (smoking status, alcohol consumption, total MVPA, sedentary behavior), BMI and chronic diseases (baseline hypertension and diabetes) [26], with adjustment for covariates the same as in the main model except for the stratified variable itself. The interaction effect of VPA to MVPA with stratified variable was assessed by introducing a multiplicative interaction term into the Cox models and the Wald test was used to calculate the *P* value for the interaction term [27].

We used the restricted cubic spline function to delineate the continuous exposure–response association between the proportion of VPA to MVPA and incident CVD and all-cause mortality with the %LGTPHCUTV9 macro, which fits restricted cubic splines to proportional hazards regression models to examine non-parametrically the relation between an exposure and the incidence rate ratio of the outcome of interest [28]. The output includes the set of *P*-values from the likelihood ratio tests for non-linearity, a linear association, and any association. We set three knots at the 50^th^, 75^th^ and 95^th^ percentiles of the proportion of VPA to MVPA.

To find the optimal combination of MPA and VPA, we also conducted an analysis to examine the joint association of MPA (0 to < 150, 150 to < 300, ≥ 300 min/week) and VPA (0 to < 75, 75 to < 150, ≥ 150 min/week) [4] with different outcomes by creating a combined variable with 3×3 mutually exclusive groups, taking the lowest MPA and VPA as reference.

Finally, to test the robustness of our main findings, we conducted sensitivity analyses by extended adjustment for hypertension, diabetes status, medical center, lipid-lowering treatment, antihypertensive medications, and diabetes medication, by extended adjustment for employment information, by conducting competing risks analyses to compare end point-specific survival between the proportion of VPA and CVD events using the Fine and Gray competing risk model [29], by excluding participants who developed CVD or died within the first two years of follow-up, and by excluding participants with missing covariates.

All statistical analyses were performed using the SAS 9.4 (SAS Institute, Cary, North Carolina, USA) and R (R Foundation for Statistical Computing, Vienna, Austria). Statistical significance was defined as a two-sided *P* < 0.05 except for the subgroup analyses, in which statistical significance was set at *P* < 0.0045 (0.05 / 11 subgroups) to account for the possible type I error generating from multi-comparisons [30].

## Results

### Characteristics at baseline

A total of 366,566 participants (46.1% were men, mean [SD] age: 56.0 [8.1] years) were included in the present study. Baseline characteristics by the proportion of VPA to MVPA were presented in Table 1. Of these, 142,476 (38.9%) reported 0% of VPA, 108,068 (29.5%) reported 0–30% of VPA, and 116,022 (31.6%) reported > 30% of total MVPA being VPA. Except for the exposure variable (MVPA, VPA and MPA) used for categorizing participants, standardized differences of baseline characteristics ranged from 0.024 to 0.184, indicating a small difference among groups (Table 1).

**Table 1:** Baseline characteristics of 366566 participants by the proportion of VPA to MVPA

	Total	VPA/MVPA			Standardized difference^*^
		0%	> 0% to ≤ 30%	> 30%	
Number	366,566	142,476	108,068	116,022	
Male, No. (%)	168,871 (46.1)	59,788 (42.0)	48,786 (45.1)	60,297 (52.0)	0.134
Age, mean (SD), years	56.0 (8.1)	56.8 (7.9)	56.4 (8.1)	54.6 (8.2)	0.184
Townsend index, mean (SD)	-1.4 (3.0)	-1.2 (3.1)	-1.6 (2.9)	-1.6 (3.0)	0.078
Education, No. (%)					0.087
College or university	131,969 (36.0)	45,062 (31.6)	39,380 (36.4)	47,527 (41.0)	
Below college	232,123 (63.3)	96,317 (67.6)	68,085 (63.0)	67,721 (58.4)	
Income (￡/year), No. (%)					0.063
< 18,000	64,384 (17.6)	29,891 (21.0)	18,340 (17.0)	16,153 (13.9)	
18,000–52,000	168,464 (46.0)	64,660 (45.4)	53,143 (49.2)	50,661 (43.7)	
≥52,000	92,444 (25.2)	30,302 (21.3)	25,057 (23.2)	37,085 (32.0)	
Race, No. (%)					0.024
White	346,972 (94.7)	134,627 (94.5)	103,346 (95.6)	108,999 (94.0)	
Others	18,554 (5.1)	7,429 (5.2)	4,424 (4.1)	6,701 (5.8)	
Smoking status, No. (%)					0.065
Never	204,756 (55.9)	77,057 (54.1)	61,002 (56.5)	66,697 (57.5)	
Former	124,485 (34.0)	47,805 (33.6)	37,255 (34.5)	39,425 (34.0)	
Current	36,389 (9.9)	17,171 (12.1)	9,589 (8.9)	9,629 (8.3)	
Alcohol consumption (g/day), No. (%)					0.072
0	25,782 (7.0)	12,490 (8.8)	6,385 (5.9)	6,907 (6.0)	
0.1–29.9	231,132 (63.1)	84,828 (59.5)	70,642 (65.4)	75,662 (65.2)	
≥30.0	50,917 (13.9)	18,674 (13.1)	14,932 (13.8)	17,311 (14.9)	
Body mass index, mean (SD), kg/m^2^	27.1 ± 4.6	27.8 ± 5.0	26.8 ± 4.3	26.7 ± 4.2	0.146
Diet quality score, mean (SD)	3.1 ± 1.4	2.9 ± 1.4	3.2 ± 1.4	3.2 ± 1.4	0.139
Hypertension, No. (%)	193,214 (52.7)	79,438 (55.8)	56,797 (52.6)	56,979 (49.1)	0.089
Diabetes, No. (%)	18,128 (5.0)	9,131 (6.4)	4,454 (4.1)	4,543 (3.9)	0.075
Lipid lowering treatment, No. (%)	47,836 (13.1)	22,088 (15.5)	13,195 (12.2)	12,553 (10.8)	0.093
Family history of CVD, No. (%)	210,062 (57.3)	83,110 (58.3)	62,503 (57.8)	64,449 (55.6)	0.037
MVPA, mean (SD), MET-minutes/week	2,716.7 (2,713.8)	1,478.2 (1,604.9)	3,588.0 (2,629.0)	3,425.9 (3,251.6)	0.594
VPA, mean (SD), MET-minutes/week	699.3 (1,216.0)	0.0 (0.0)	555.5 (555.9)	1,691.9 (1,667.0)	1.254
MPA, mean (SD), MET-minutes/week	953.1 (1,229.3)	569.5 (971.8)	1,494.3 (1,448.6)	920.1 (1,098.1)	0.512
Sedentary behavior, mean (SD), hours/day	4.5 (2.5)	4.6 (2.6)	4.3 (2.4)	4.4 (2.6)	0.104

Abbreviations: CVD, cardiovascular disease; MET, metabolic equivalent of task; MPA, moderate physical activity; MVPA, moderate-to-vigorous intensity physical activity; VPA, vigorous physical activity. VPA/MVPA, the proportion of VPA to MVPA

Data are presented as means ± SD for continuous variables or n (percentages) for categorical variables

The number of missing data were 2,474 (0.7%), 41,274 (11.3%), 1,040 (0.3%), 462 (0.1%), 936 (0.3%), 58,735 (16%), 1,400 (0.4%) and 64 (0.02%) for education, income, race, Townsend index, smoking status, alcohol consumption, BMI and sedentary behavior

^*^Average standardized difference of three standardized differences from group comparisons (namely group 0% VS > 0% to ≤ 30%, group > 0% to ≤ 30% VS > 30%, group 0% VS > 30%)

### Association of proportion of VPA to MVPA with incident CVD and all-cause mortality

During a median 11.8 years of follow-up, we documented 31,894 incident CVD cases (including 23,657 CHD cases, 6,905 HF cases and 6,293 stroke cases) and 19,823 total deaths (including 3,359 CVD deaths). In the fully adjusted models, compared with participants with 0% of VPA to MVPA, the fully-adjusted HRs for participants with 0% to 30% of VPA to MVPA were 0.88 (95% CI, 0.86–0.91) and 0.81 (95% CI, 0.78–0.84), respectively for incident CVD and all-cause mortality. The corresponding HRs were 0.89 (95% CI, 0.86–0.92) and 0.82 (95% CI, 0.79–0.85) for those reporting more than 30% of VPA to MVPA (*P*_trend_ < 0.001, Table 2). Similar associations were observed across different subtypes of CVD and mortality, the corresponding HRs (95% CIs) were 0.89 (0.86–0.93), 0.78 (0.72–0.84) and 0.86 (0.77–0.96) for incident CHD, HF and CVD mortality, respectively, while non-significant association was seen for incident stroke (HR [95% CI]: 0.93 [0.86–1.00]; Table 2). The results did not change materially by further adjustments for conventional CVD risk factors or employment, excluding subjects developed CVD or died within 2 years, using the competing risk model, or excluding participants with missing covariates. The corresponding HRs for incident CVD were 0.89 (0.87–0.92), 0.88 (0.85–0.91), 0.89 (0.86–0.92), 0.88 (0.86–0.91), 0.90 (0.86–0.93), respectively (see Additional file 4).

**Table 2:** Association of the proportion of VPA to MVPA with incident CVD and all-cause mortality

	VPA/MVPA			*P* trend^*^
	0%	> 0% to ≤ 30%	> 30%	
Incident CVD				
Cases/10,000 person-years	90	73	66	
Model 1	1 [ref.]	0.80 (0.78–0.82)	0.79 (0.77–0.81)	< 0.001
Model 2	1 [ref.]	0.88 (0.86–0.91)	0.89 (0.86–0.92)	< 0.001
CHD				
Cases/10,000 person-years	66	53	49	
Model 1	1 [ref.]	0.79 (0.77–0.82)	0.79 (0.77–0.82)	< 0.001
Model 2	1 [ref.]	0.87 (0.84–0.90)	0.89 (0.86–0.93)	< 0.001
HF				
Cases/10,000 person-years	21	14	12	
Model 1	1 [ref.]	0.69 (0.66–0.74)	0.67 (0.63–0.71)	< 0.001
Model 2	1 [ref.]	0.83 (0.78–0.88)	0.78 (0.72–0.84)	< 0.001
Stroke				
Cases/10,000 person-years	17	14	13	
Model 1	1 [ref.]	0.86 (0.81–0.92)	0.86 (0.81–0.91)	< 0.001
Model 2	1 [ref.]	0.93 (0.88–0.99)	0.93 (0.86–1.00)	0.064
All-cause mortality				
Cases/10,000 person-years	58	41	37	
Model 1	1 [ref.]	0.72 (0.69–0.74)	0.71 (0.69–0.74)	< 0.001
Model 2	1 [ref.]	0.81 (0.78–0.84)	0.82 (0.79–0.85)	< 0.001
CVD mortality				
Cases/10,000 person-years	10	7	7	
Model 1	1 [ref.]	0.65 (0.60–0.71)	0.73 (0.67–0.79)	< 0.001
Model 2	1 [ref.]	0.77 (0.70–0.84)	0.86 (0.77–0.96)	0.019

Abbreviations: CVD, cardiovascular disease; CHD, coronary heart disease; HF, heart failure; MVPA, moderate-to-vigorous intensity physical activity; VPA, vigorous physical activity. VPA/MVPA, the proportion of VPA to MVPA

Model 1 was adjusted for age and sex (male or female)

Model 2 was additionally adjusted for education (college/university or below college), income (< 18,000 or 18,000–52,000 or ≥ 52,000 ￡/year), race (white or others), Townsend index, smoking status (never or current or former), alcohol consumption (0 or 0.1–30 or ≥ 30 g/day), sedentary behavior (hours/day), MVPA (MET-hours/week), BMI (kg/m^2^), diet quality score and family history of CVD

^*^The tests for *P* trends were conducted by assigning the median value as the continuous variable for each group of VPA to MVPA (0%, > 0% to ≤ 30% and > 30%)

In the subgroup analysis, we found a significant interaction effect of VPA to MVPA and age on the incident CVD and all-cause mortality (all *P*_interaction_ < 0.005), the associations of VPA with incident CVD and all-cause mortality tended to be more obvious among participants aged less than 65 years old, the HRs (95% CIs) of > 30% of VPA to MVPA were 0.79 (0.76–0.82) and 0.72 (0.69–0.76), respectively, for incident CVD and all-cause mortality. Similarly, we observed significant interaction of VPA to MVPA and smoking on the incident CVD and all-cause mortality (all *P*_interaction_ < 0.001), stronger association were seen among current smokers than non-smokers, the corresponding HRs (95% CIs) were 0.81 (0.78–0.85) and 0.75 (0.71–0.78), respectively. In addition, the health benefits were consistent across sex, education, Townsend index, alcohol consumption, BMI, hypertension, diabetes, total MVPA and daily sedentary hours. (*P*_interaction_ > 0.005; see Additional file 5 and 6).

### Exposure–response association of the proportion of VPA to MVPA with incident CVD and all-cause mortality

**Fig. 1:**
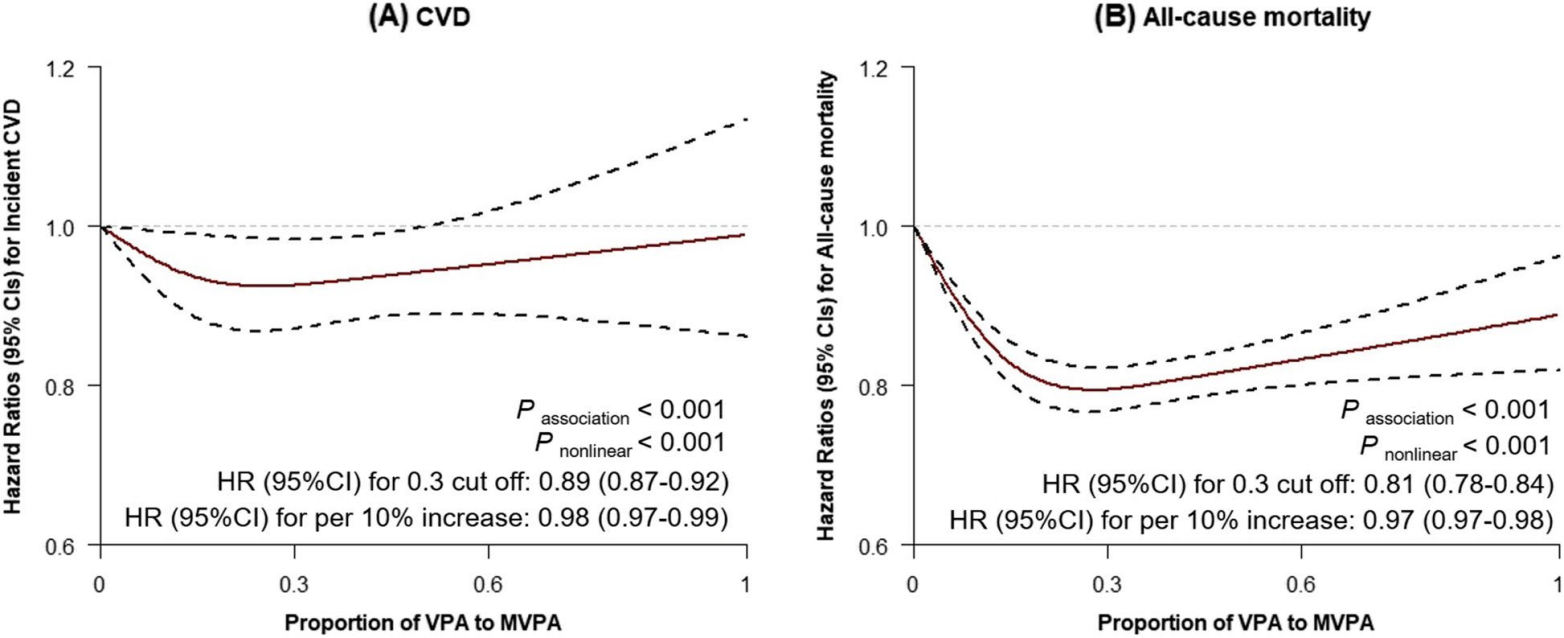
Associations of VPA proportion with incident CVD and all-cause mortality. Abbreviations: CVD, cardiovascular disease; MVPA, moderate-to-vigorous intensity physical activity; VPA, vigorous physical activity. Models were adjusted for age, sex, education, income, race, Townsend index, smoking status, alcohol consumption, BMI, sedentary behavior, MVPA, diet quality score and family history of CVD

The cubic spline showed an L-shape in both incident CVD and all-cause mortality (*P*_association_ < 0.001). Cubic spline showed a decrease in risks of incident CVD with increasing proportion of VPA to MVPA before 30% of VPA to MVPA, and plateaued thereafter without upward trend (Fig. 1A). For all-cause mortality, similar pattern of association was observed, but the mortality risk reached a nadir at about 30% of VPA to MVPA, and then rose slowly but never reached the level of no VPA (Fig. 1B). The maximum risk reduction occurred at approximately 30% of VPA to MVPA, with the HRs (95% CIs) of 0.89 (0.87–0.92) and 0.81 (0.78–0.84), respectively, for incident CVD and all-cause mortality (Fig. 1). Consistent associations were seen for CVD subtypes and CVD mortality (*P*_association_ < 0.05). The corresponding HRs (95% CIs) at 30% of VPA to MVPA were 0.87 (0.85–0.90), 0.81 (0.77–0.87), 0.92 (0.86–0.98), and 0.79 (0.72–0.86), respectively, for incident CHD, HF, stroke, and CVD mortality (see Additional file 7 and 8).

### Joint associations of MPA and VPA with incident CVD and all-cause mortality

Compared with those who reported the least MPA and VPA (0 to < 150 min of MPA and 0 to < 75 min of VPA), those performing 150 to 300 min of MPA and ≥ 150 min of VPA per week had the lowest risk of incident CVD (HR [95% CI], 0.87 [0.79–0.95]) and all-cause mortality (HR [95% CI], 0.71 [0.63–0.80]; Table 3). Similar results were observed for incident CHD (HR [95% CI], 0.86 [0.77–0.95]). For incident HF and CVD mortality, the optimum combination of MPA and VPA was 150 to 300 min/week of MPA and 75–150 min/week of VPA, the corresponding HRs (95% CIs) were 0.77 (0.65–0.92) and 0.72 (0.57–0.93), respectively (see Additional files 9 and 10).

**Table 3:** Joint associations of MVPA with incident CVD and all-cause mortality

	MPA, minutes/week		
Incident CVD	0 to < 150	150 to < 300	≥300
VPA, minutes/week			
0 to < 75	1 [Ref.]	0.98 (0.95–1.02)	0.98 (0.95–1.01)
75 to < 150	0.92 (0.83–1.02)	0.90 (0.84–0.97)	0.93 (0.89–0.98)
≥150	0.93 (0.83–1.05)	0.87 (0.79–0.95)	0.95 (0.92–0.99)
All-cause mortality			
VPA, minutes/week			
0 to < 75	1 [Ref.]	0.93 (0.89–0.97)	0.87 (0.84–0.91)
75 to < 150	0.86 (0.75–0.99)	0.75 (0.68–0.83)	0.77 (0.72–0.82)
≥150	0.78 (0.66–0.92)	0.71 (0.63–0.80)	0.78 (0.75–0.82)

Abbreviations: CVD, cardiovascular disease; MVPA, moderate-to-vigorous intensity physical activity; MPA, moderate physical activity; VPA, vigorous physical activity

Models were adjusted for age, sex, education, income, race, Townsend index, smoking status, alcohol consumption, BMI, sedentary behavior, diet quality score and family history of CVD

## Discussion

In this large prospective cohort study of UK adults, performing VPA was associated with lower risks of incident CVD and all-cause mortality. Participants reporting 30% of VPA appeared to experience the lowest risks of incident CVD and all-cause mortality. Moreover, we found that participants performing 150–300 min of MPA and ≥ 150 min of VPA per week had the lowest risks of CVD and all-cause mortality. To our best knowledge, this is the first prospective study investigating the association of the proportion of VPA to MVPA as well as the joint association of MPA and VPA with incident CVD.

### Comparison with other studies

In the present study, we found participants who performed VPA had significantly lower risks of all-cause mortality than no VPA, with the lowest risk occurring at about 30% VPA to MVPA, which was generally in line with previous studies [6, 17]. The study of 11 cohorts reported similar magnitude of HR (0.84) in association between VPA and all-cause mortality among those performed 0%-30% and 30% or more of VPA [6]. Another cohort study of 204,542 middle-aged and older Australians reported that 0%-30% of VPA was associated with a 9% reduction of all-cause mortality (HR, 0.91 [95% CI, 0.84–0.98]), while no additional benefits in lowering mortality risk were seen among those with 30% or more of VPA [17], which was aligned with our study. The reason might be that high levels of VPA was positively associated with oxidative stress [31, 32] which might counteract the beneficial effects from physical activity.

Several prospective cohort studies have investigated the associations of VPA versus MPA with CVD mortality [5, 6, 33]. A prospective study of 403,681 US adults (mean age, 42.8 years) [5] identified 17% lower risks of CVD mortality among those performing 50%-75% and 75%-100% of VPA to MVPA. Another study of 7,979 men and 38,671 women from the Harvard Alumni Health Study only identified a 26% lower risk of CVD mortality (HR, 0.74 [95% CI, 0.58–0.93]) among men who performed 25%-50% of VPA to MVPA [33]. However, a study of 64,913 adults from 11 population cohorts and observed non-significant associations of VPA with CVD mortality (HR [95% CI] of 0%-30% VPA and ≥ 30% VPA, 0.83 [0.57–1.18] and 0.84 [0.68–1.04]) [6].The present study found new evidence that a higher proportion of VPA is associated with lower risks of CVD mortality compared with MPA, while the US study [5] only observed significant associations among participants with 50%-99% of VPA for CVD mortality, which might partly be due to the small number of deaths collected in the 0%-50% group (306 CVD deaths). Notably, the present study identified for the first time that 0%-30% and ≥ 30% VPA to MVPA were associated with 12% and 11% lower risk of incident CVD, which were not examined in previous studies. The possible reason that VPA was associated with larger cardiovascular benefits might be that VPA might increase peak oxygen uptake thus improving cardiorespiratory fitness [34].

The inverse association was less obvious among older adults than their younger counterparts. This might be because older adults are more likely to suffer from multiple comorbidities, and their cardiac condition may also limit their capacity in performing VPA [35, 36]. Another interesting finding of our study was that current smokers experienced more health benefits than non-smokers by performing a higher volume of VPA. A previous study of 17,944 British men also found that smoked men who engaged in VPA experienced approximately half of the CHD incidence than smokers with no VPA (CHD incidence: 4.9 VS 9.7) [37]. The current study provides evidence that guideline on physical activity should be specified for target pollution such as middle-aged adults and smokers, and highlights the need for more epidemiological studies to validate these findings in different population [38].

### Strengths and limitations

The major strengths include the large sample size, approximately 12 years of follow-up, accounting for a total amount of MVPA, examining subtypes of CVD in addition to overall CVD, and exploring the joint association of MPA and VPA for the first time to our knowledge. This study has several limitations. First, information on physical activity was obtained once by self-report and might be inaccurate in differentiating different intensities of physical activity. Second, the UK Biobank sample was not representative of the UK population and more than 90% of the participants included were white, therefore the generalization of our results to other population should be cautious. Finally, the possibility of reverse causation still existed because participants with underlying diseases were less likely to perform VPA and more likely to have a CVD event or die; however, we adjusted for pre-existing diseases and medications at baseline, excluded events or deaths in the first 2 years of follow-up, and the results were largely unchanged.

## Conclusions

The current study indicated lower risks of incident CVD and all-cause mortality at 0% ~ 30% of VPA to MVPA, no additional benefit at more than 30% of VPA to MVPA. To maximum population health, we recommended a combination of 150–300 min/week of MPA and ≥ 150 min/week of VPA for middle-aged and older adults.

## Supplementary Information

**Additional file 1.**

**Additional file 2.**

**Additional file 3.**

**Additional file 4.**

**Additional file 5**.

**Additional file 6**.

**Additional file 7.**

**Additional file 8.**

**Additional file 9.**

**Additional file 10.**

## Data Availability

All the data were extracted from the UK Biobank, which are publicly available to qualified researchers on application to the UK Biobank (www.ukbiobank.ac.uk). The code is available from the corresponding author upon reasonable request.

## Abbreviations

BMI: Body mass index
CHD: Coronary heart disease
CIs: Confidence intervals
CVD: Cardiovascular disease
HES: Hospital diagnoses from the National Health Service
HF: Heart failure
HR: Hazard ratios
MET: Metabolic equivalent task
MPA: Moderate physical activity
PA: Physical activity
VPA: Vigorous physical activity
