# Supplementary material for: Associations of physical activity intensity with incident cardiovascular diseases and mortality among 366,566 UK adults
Source: Int J Behav Nutr Phys Act. 2022 Dec 13;19:151. doi: 10.1186/s12966-022-01393-y (PMC9745930; doi:10.1186/s12966-022-01393-y)

Associations of physical activity intensity with incident cardiovascular diseases and mortality among 366,566 UK adults

Additional file 1: Inclusion and exclusion flow chart.


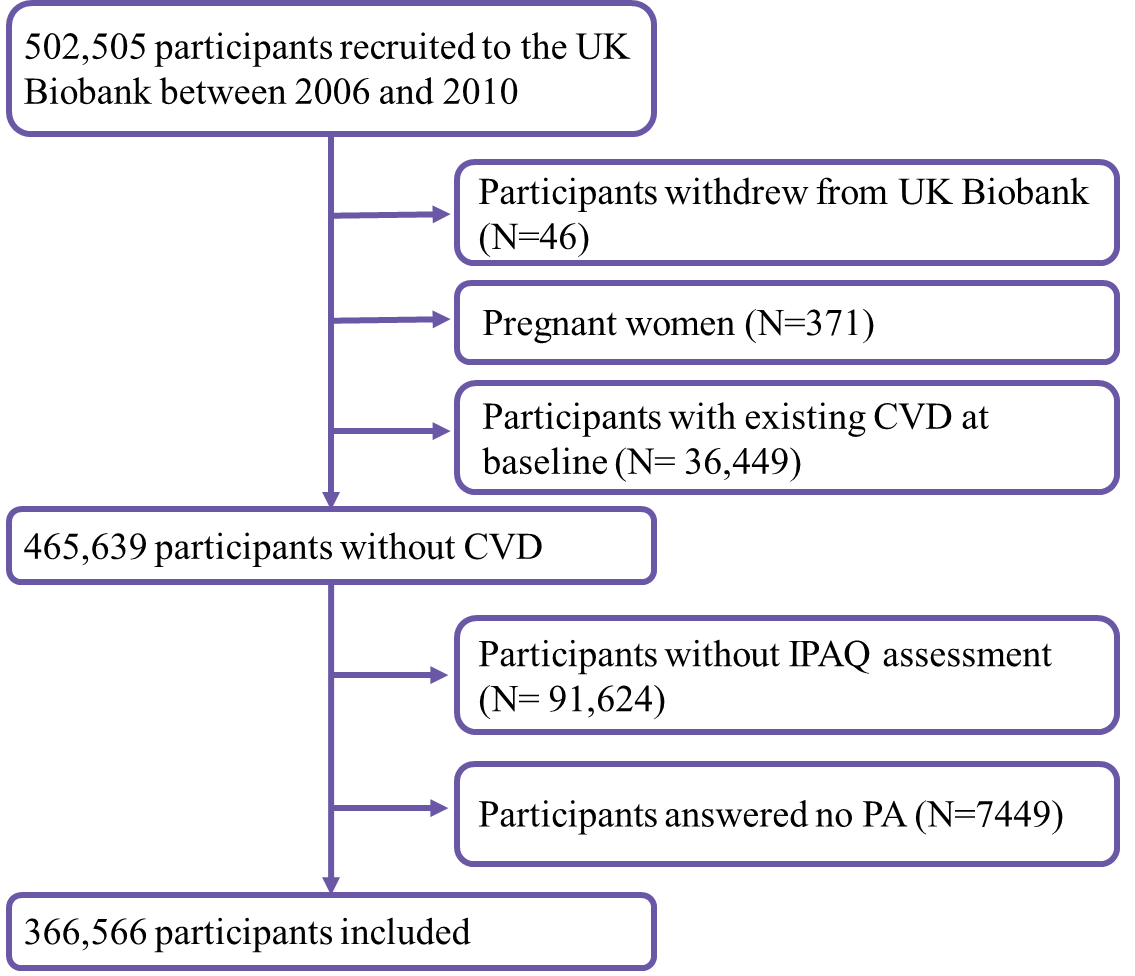

Supplement: Supplementary file 1 — Additional file 1. [file 12966_2022_1393_MOESM1_ESM.docx]
