# Supplementary material for: Associations of physical activity intensity with incident cardiovascular diseases and mortality among 366,566 UK adults
Source: Int J Behav Nutr Phys Act. 2022 Dec 13;19:151. doi: 10.1186/s12966-022-01393-y (PMC9745930; doi:10.1186/s12966-022-01393-y)
Supplement: Supplementary file 2 — Additional file 2. [file 12966_2022_1393_MOESM2_ESM.docx]

Associations of physical activity intensity with incident cardiovascular diseases and mortality among 366,566 UK adults

| Additional file 2: Comparison of participants before and after exclusion of participants without physical activity information. | | | |
| --- | --- | --- | --- |
|  | Before exclusion | After exclusion | Standardized difference |
| Male, No. (%) | 205,388 (44.1) | 168,871 (46.1) | 0.039 |
| Age, means (SD), years | 56.2 (8.1) | 56.0 (8.1) | 0.025 |
| Townsend index | -1.4 (3.1) | -1.4 (3.0) | 0.030 |
| Education, No. (%) |  |  | 0.056 |
| College or university | 153,363 (32.9) | 131,969 (36.0) |  |
| Below college | 303,162 (65.1) | 232,123 (63.3) |  |
| Income (￡/year), No. (%) |  |  | 0.077 |
| <18,000 | 84,932 (18.2) | 64,384 (17.6) |  |
| 18,000-52,000 | 205,258 (44.1) | 168,464 (46.0) |  |
| ≥52,000 | 105,323 (22.6) | 92,444 (25.2) |  |
| Race, No. (%) |  |  | 0.014 |
| White | 438,199 (94.1) | 346,972 (94.7) |  |
| Others | 24,946 (5.4) | 18,554 (5.1) |  |
| Smoking status, No. (%) |  |  | 0.009 |
| Never | 258,798 (55.6) | 204,756 (55.9) |  |
| Former | 48,232 (10.4) | 36,389 (9.9) |  |
| Current | 156,000 (33.5) | 124,485 (34.0) |  |
| Alcohol consumption (g/day), No. (%) |  |  | 0.034 |
| 0 | 35,876 (7.7) | 25,782 (7.0) |  |
| 0.1-29.9 | 287,262 (61.7) | 231,132 (63.1) |  |
| ≥30.0 | 61,066 (13.1) | 50,917 (13.9) |  |
| BMI, means (SD), kg/m^2^ | 27.3 (4.8) | 27.1 (4.6) | 0.035 |
| Diet quality score, means (SD) | 3.1 (1.4) | 3.1 (1.4) | 0.017 |
| Hypertension, No. (%) | 250,287 (53.8) | 193,214 (52.7) | 0.021 |
| Diabetes, No. (%) | 24,634 (5.3) | 18,128 (5.0) | 0.016 |
| Cancer, No. (%) | 51,183 (11.0) | 39,546 (10.8) | 0.007 |
| Lipid lowering treatment, No. (%) | 62,925 (13.5) | 47,836 (13.1) | 0.014 |
| Family history of CVD, No. (%) | 266,653 (57.3) | 210,062 (57.3) | 0.001 |
| MVPA, means (SD), MET-minutes/week | 2,662.6 (2,713.3) | 2,716.7 (2,713.8) | 0.020 |
| VPA, means (SD), MET-minutes/week | 685.4 (1,207.8) | 699.3 (1,216.0) | 0.011 |
| MPA, means (SD), MET-minutes/week | 934.1 (1,224.3) | 953.1 (1,229.3) | 0.015 |
| Sedentary behavior, means (SD), hours/day | 4.5 (2.6) | 4.5 (2.5) | 0.008 |
| Data are means (SD) (for continuous variables) or percentages (for categorical variables). | | | |
