# Supplementary material for: Associations of physical activity intensity with incident cardiovascular diseases and mortality among 366,566 UK adults
Source: Int J Behav Nutr Phys Act. 2022 Dec 13;19:151. doi: 10.1186/s12966-022-01393-y (PMC9745930; doi:10.1186/s12966-022-01393-y)
Supplement: Supplementary file 3 — Additional file 3. [file 12966_2022_1393_MOESM3_ESM.docx]

Associations of physical activity intensity with incident cardiovascular diseases and mortality among 366,566 UK adults

| Additional file 3: Definition of each component of a diet quality score. | | | |
| --- | --- | --- | --- |
|  | Goal (1 point) | One serving equals to | Field |
| Fruits | ≥ 3 servings/day | 1 piece of fresh fruit  5 pieces of dried fruit | 1309, 1319 |
| Vegetables  (excluding potatoes) | ≥ 3 servings/day | 3 heaped tablespoons | 1289, 1299 |
| Whole grains | ≥ 3 servings/day | 1 slice of whole-grain bread  1 cup of whole-grain cereal | 1438, 1448, 1458, 1468 |
| Vegetable oil | ≥ 2 servings/day | Vegetable oil based spread (Flora Pro-Active/Benecol, Soft (tub) margarine, Olive oil based spread, or Polyunsaturated/sunflower oil based spread) in combination with eating at least 2 slices of bread | 1428, 2654, 1438 |
| Fish | ≥ 2 servings/week | Once/week | 1329, 1339 |
| Dairy | ≥ 2 servings/day | 1 cup/day if consumption any type of milk  1 piece of cheese | 1408, 1418 |
| Refined grains | ≤ 2 servings/day | 1 slice of bread  1 bowl of cereal | 1438, 1448, 1458, 1468 |
| Unprocessed meats | ≤ 2 servings/week | Once/week (including poultry, beef, lamb, and pork) | 1359, 1369, 1379, 1389 |
| Processed meats | ≤ 1 servings/week | Once/week | 1349 |
| Sugar-sweetened beverages | Don’t drink | Only 0 serving was possible here | 6144 |
