# Supplementary material for: Associations of physical activity intensity with incident cardiovascular diseases and mortality among 366,566 UK adults
Source: Int J Behav Nutr Phys Act. 2022 Dec 13;19:151. doi: 10.1186/s12966-022-01393-y (PMC9745930; doi:10.1186/s12966-022-01393-y)
Supplement: Supplementary file 4 — Additional file 4. [file 12966_2022_1393_MOESM4_ESM.docx]

Associations of physical activity intensity with incident cardiovascular diseases and mortality among 366,566 UK adults

| Additional file 4: Association of the proportion of VPA to MVPA with incident CVD and all-cause mortality in the sensitivity analyses. | | | | | | | | |
| --- | --- | --- | --- | --- | --- | --- | --- | --- |
|  | No. of cases (%) | | Main model | With extended adjustment for conventional CVD risk factors^a^ | Further adjust for employment information | Using Fine & Gray models for competing risk analyses | Excluding subjects who developed CVD or died within 2 years | Excluding subjects with missing covariates |
| **Incident CVD** |  | |  |  |  |  |  |  |
| 0% | 14325 (0.1) | | Ref. | Ref. | Ref. | Ref. | Ref. | Ref. |
| >0% to ≤ 30% | 7392 (0.08) | | 0.88 (0.86-0.91) | 0.90 (0.87-0.92) | 0.88 (0.86-0.91) | 0.89 (0.86-0.91) | 0.89 (0.86-0.91) | 0.88 (0.85-0.91) |
| >30% | 6427 (0.08) | | 0.89 (0.86-0.92) | 0.89 (0.87-0.92) | 0.88 (0.85-0.91) | 0.88 (0.86-0.91) | 0.89 (0.86-0.92) | 0.90 (0.86-0.93) |
| **CHD** |  | |  |  |  |  |  |  |
| 0% | 10604 (0.07) | | Ref. | Ref. | Ref. | Ref. | Ref. | Ref. |
| >0% to ≤ 30% | 5412 (0.06) | | 0.87 (0.84-0.90) | 0.88 (0.85-0.91) | 0.87 (0.84-0.90) | 0.87 (0.84-0.90) | 0.87 (0.84-0.90) | 0.86 (0.83-0.90) |
| >30% | 4806 (0.06) | | 0.89 (0.86-0.93) | 0.89 (0.86-0.92) | 0.88 (0.85-0.91) | 0.88 (0.85-0.91) | 0.89 (0.86-0.92) | 0.89 (0.85-0.94) |
| **HF** |  | |  |  |  |  |  |  |
| 0% | 3424 (0.02) | | Ref. | Ref. | Ref. | Ref. | Ref. | Ref. |
| >0% to ≤ 30% | 1477 (0.02) | | 0.83 (0.78-0.88) | 0.84 (0.79-0.90) | 0.83 (0.78-0.88) | 0.83 (0.78-0.88) | 0.84 (0.79-0.89) | 0.82 (0.77-0.89) |
| >30% | 1266 (0.02) | | 0.78 (0.72-0.84) | 0.82 (0.77-0.87) | 0.80 (0.75-0.85) | 0.81 (0.76-0.86) | 0.82 (0.77-0.88) | 0.77 (0.71-0.85) |
| **Stroke** |  | |  |  |  |  |  |  |
| 0% | 2762 (0.02) | | Ref. | Ref. | Ref. | Ref. | Ref. | Ref. |
| >0% to ≤ 30% | 1515 (0.02) | | 0.93 (0.88-0.99) | 0.95 (0.89-1.01) | 0.93 (0.87-0.99) | 0.93 (0.88-0.99) | 0.93 (0.87-1.00) | 0.93 (0.86-1.00) |
| >30% | 1278 (0.02) | | 0.93 (0.86-1.00) | 0.95 (0.89-1.01) | 0.93 (0.87-0.99) | 0.93 (0.87-1.00) | 0.93 (0.87-1.00) | 0.94 (0.86-1.03) |
| **All-cause mortality** | |  |  |  |  |  |  |  |
| 0% | 9622 (0.07) | | Ref. | Ref. | Ref. | Ref. | Ref. | Ref. |
| >0% to ≤ 30% | 4371 (0.05) | | 0.81 (0.78-0.84) | 0.82 (0.80-0.86) | 0.81 (0.78-0.84) | - | 0.82 (0.79-0.85) | 0.81 (0.77-0.84) |
| >30% | 3676 (0.04) | | 0.82 (0.79-0.85) | 0.84 (0.81-0.87) | 0.82 (0.79-0.85) | - | 0.84 (0.80-0.87) | 0.82 (0.79-0.86) |
| **CVD mortality** |  | |  |  |  |  |  |  |
| 0% | 1642 (0.01) | | Ref. | Ref. | Ref. | Ref. | Ref. | Ref. |
| >0% to ≤ 30% | 700 (0.01) | | 0.73 (0.67-0.79) | 0.78 (0.71-0.85) | 0.76 (0.70-0.83) | 0.77 (0.70-0.84) | 0.76 (0.69-0.84) | 0.74 (0.67-0.83) |
| >30% | 617 (0.01) | | 0.86 (0.77-0.96) | 0.90 (0.82-0.98) | 0.87 (0.79-0.95) | 0.87 (0.80-0.95) | 0.87 (0.79-0.95) | 0.91 (0.80-1.03) |
| Abbreviations: CVD, cardiovascular disease; CHD, coronary heart disease; HF, heart failure. MVPA, moderate-to-vigorous intensity physical activity; VPA, vigorous physical activity. VPA/MVPA, the proportion of VPA to MVPA.  ^a^Models were additionally adjusted for hypertension, diabetes status, lipid-lowering treatment, antihypertensive medications, diabetes medication and medical center. | | | | | | | | |
