# Supplementary material for: Associations of physical activity intensity with incident cardiovascular diseases and mortality among 366,566 UK adults
Source: Int J Behav Nutr Phys Act. 2022 Dec 13;19:151. doi: 10.1186/s12966-022-01393-y (PMC9745930; doi:10.1186/s12966-022-01393-y)
Supplement: Supplementary file 5 — Additional file 5. [file 12966_2022_1393_MOESM5_ESM.docx]

Associations of physical activity intensity with incident cardiovascular diseases and mortality among 366,566 UK adults

Additional file 5: The association between VPA to MVPA and incident CVD stratified by risk factors.


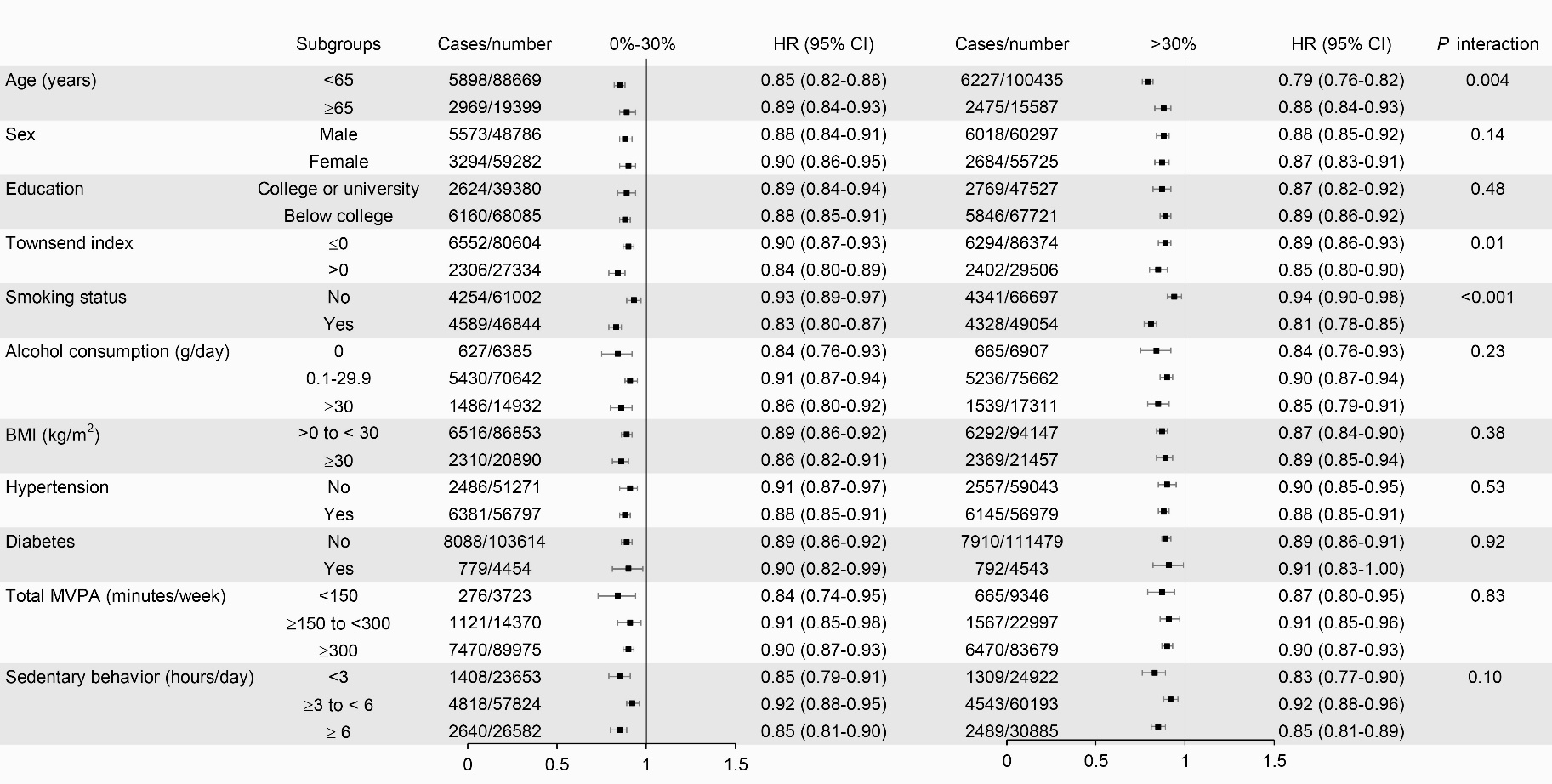


Model was adjusted for age, sex, education, income, race, Townsend index, smoking status, alcohol consumption, sedentary behavior, MVPA, BMI, diet quality score and family history of CVD.

The reference group was set at 1
