# Supplementary material for: Associations of physical activity intensity with incident cardiovascular diseases and mortality among 366,566 UK adults
Source: Int J Behav Nutr Phys Act. 2022 Dec 13;19:151. doi: 10.1186/s12966-022-01393-y (PMC9745930; doi:10.1186/s12966-022-01393-y)
Supplement: Supplementary file 7 — Additional file 7. [file 12966_2022_1393_MOESM7_ESM.docx]

Associations of physical activity intensity with incident cardiovascular diseases and mortality among 366,566 UK adults

Additional file 7: Restrict cubic spline for associations of the proportion of VPA to MVPA with incident CVD subtypes。


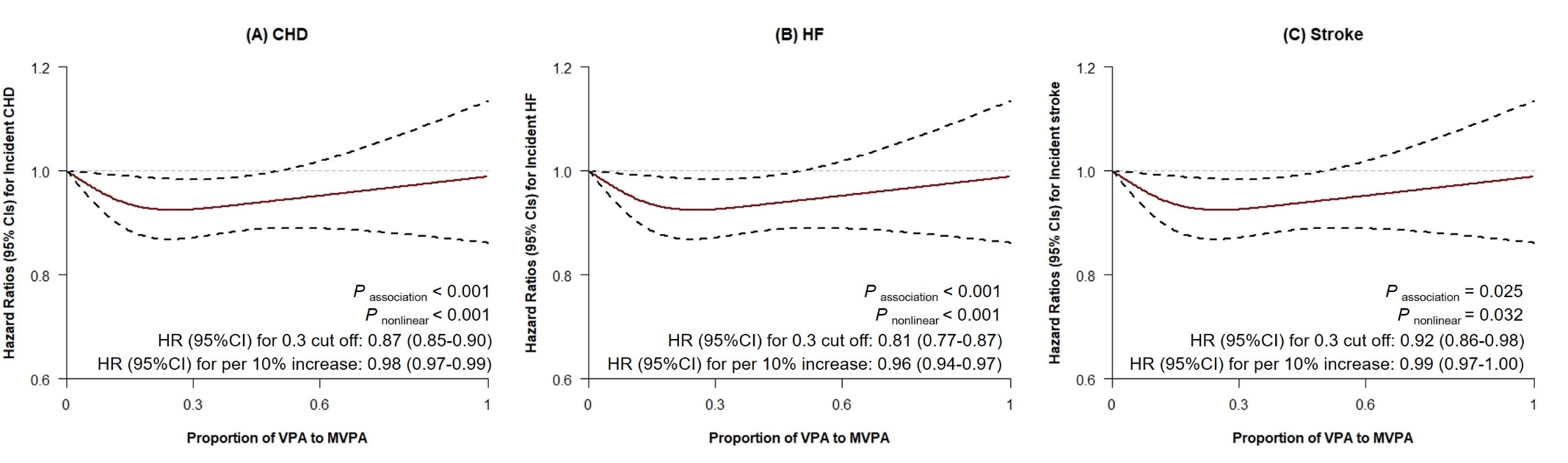


1. CHD; (B) HF; (C) Stroke

Models were adjusted for age, sex, education, income, race, Townsend index, smoking status, alcohol consumption, BMI, sedentary behaviour, diet quality score and family history of CVD
