# Supplementary material for: Associations of physical activity intensity with incident cardiovascular diseases and mortality among 366,566 UK adults
Source: Int J Behav Nutr Phys Act. 2022 Dec 13;19:151. doi: 10.1186/s12966-022-01393-y (PMC9745930; doi:10.1186/s12966-022-01393-y)
Supplement: Supplementary file 8 — Additional file 8. [file 12966_2022_1393_MOESM8_ESM.docx]

Associations of physical activity intensity with incident cardiovascular diseases and mortality among 366,566 UK adults

Additional file 8: Restrict cubic spline for associations of the proportion of VPA to MVPA with CVD mortality.


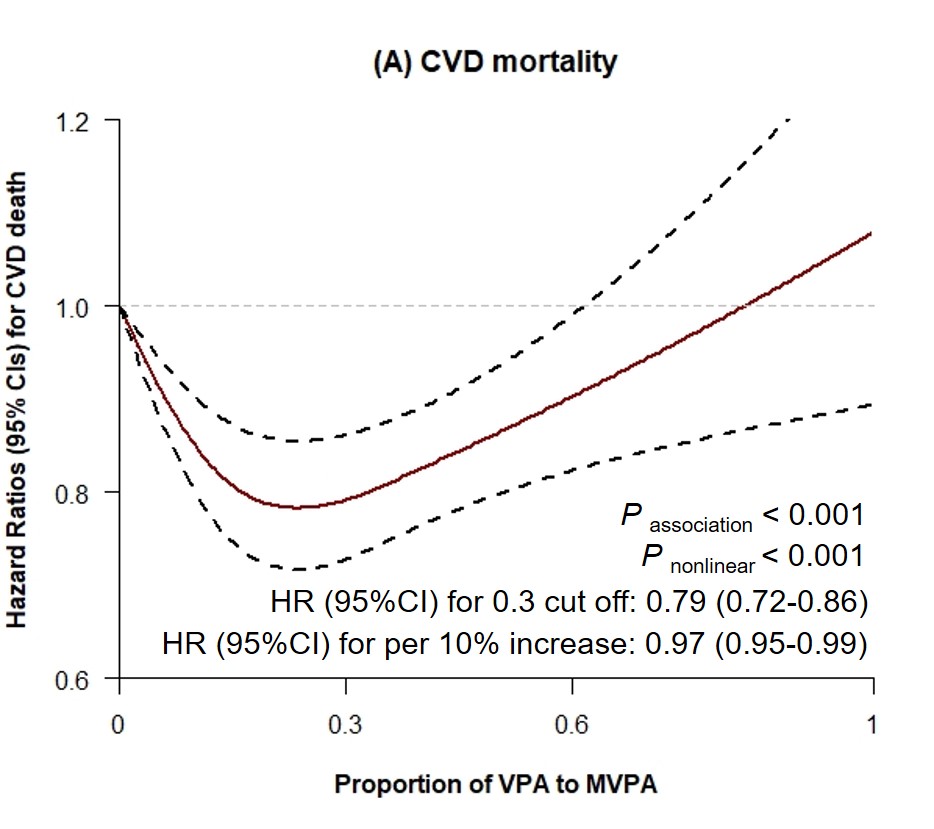


Models were adjusted for age, sex, education, income, race, Townsend index, smoking status, alcohol consumption, BMI, sedentary behavior, diet quality score and family history of CVD.
