# Supplementary material for: Associations of physical activity intensity with incident cardiovascular diseases and mortality among 366,566 UK adults
Source: Int J Behav Nutr Phys Act. 2022 Dec 13;19:151. doi: 10.1186/s12966-022-01393-y (PMC9745930; doi:10.1186/s12966-022-01393-y)
Supplement: Supplementary file 9 — Additional file 9. [file 12966_2022_1393_MOESM9_ESM.docx]

Associations of physical activity intensity with incident cardiovascular diseases and mortality among 366,566 UK adults

| Additional file 9: Adjusted hazard ratios for joint associations of MPA and VPA with incident CVD subtypes. | | | |
| --- | --- | --- | --- |
|  | MPA, minutes/week | | |
| Incident CHD | 0 to < 150 | 150 to < 300 | ≥ 300 |
| VPA, minutes/week |  |  |  |
| 0 to < 75 | 1 [Ref.] | 0.98 (0.94-1.03) | 0.98 (0.94-1.02) |
| 75 to <150 | 0.92 (0.82-1.04) | 0.92 (0.85-1.00) | 0.93 (0.89-0.98) |
| ≥ 150 | 0.90 (0.78-1.03) | 0.86 (0.77-0.95) | 0.96 (0.92-1.01) |
| Incident HF |  |  |  |
| VPA, minutes/week |  |  |  |
| 0 to < 75 | 1 [Ref.] | 0.93 (0.86-1.01) | 0.94 (0.88-1.00) |
| 75 to <150 | 0.89 (0.70-1.13) | 0.77 (0.65-0.92) | 0.79 (0.71-0.87) |
| ≥ 150 | 0.87 (0.65-1.15) | 0.81 (0.67-0.99) | 0.88 (0.81-0.95) |
| Incident stroke |  |  |  |
| VPA, minutes/week |  |  |  |
| 0 to < 75 | 1 [Ref.] | 0.99 (0.91-1.08) | 0.97 (0.90-1.04) |
| 75 to <150 | 0.89 (0.69-1.14) | 0.87 (0.74-1.03) | 0.98 (0.88-1.08) |
| ≥ 150 | 1.13 (0.88-1.46) | 0.94 (0.77-1.14) | 0.97 (0.89-1.06) |
| (A) CHD; (B) HF; (C) Stroke  Models were adjusted for age, sex, education, income, race, Townsend index, smoking status, alcohol consumption, BMI, sedentary behavior, diet quality score and family history of CVD. | | | |
