# Supplementary material for: Associations of physical activity intensity with incident cardiovascular diseases and mortality among 366,566 UK adults
Source: Int J Behav Nutr Phys Act. 2022 Dec 13;19:151. doi: 10.1186/s12966-022-01393-y (PMC9745930; doi:10.1186/s12966-022-01393-y)
Supplement: Supplementary file 10 — Additional file 10. [file 12966_2022_1393_MOESM10_ESM.docx]

Associations of physical activity intensity with incident cardiovascular diseases and mortality among 366,566 UK adults

| Additional file 10: Adjusted hazard ratios for joint associations of MPA and VPA with CVD mortality. | | | |
| --- | --- | --- | --- |
|  | MPA, minutes/week | | |
| Incident CVD | 0 to < 150 | 150 to < 300 | ≥ 300 |
| VPA, minutes/week |  |  |  |
| 0 to < 75 | 1 [Ref.] | 0.95 (0.84-1.06) | 0.88 (0.80-0.98) |
| 75 to <150 | 0.97 (0.70-1.34) | 0.72 (0.57-0.93) | 0.80 (0.69-0.93) |
| ≥ 150 | 1.01 (0.70-1.46) | 0.92 (0.71-1.20) | 0.89 (0.79-1.01) |

Models were adjusted for age, sex, education, income, race, Townsend index, smoking status, alcohol consumption, BMI, sedentary behavior, diet quality score and family history of CVD.
